# Monitoring microseismicity of the Hengill Geothermal Field in Iceland

**DOI:** 10.1038/s41597-022-01339-w

**Published:** 2022-05-19

**Authors:** Francesco Grigoli, John F. Clinton, Tobias Diehl, Philipp Kaestli, Luca Scarabello, Thorbjorg Agustsdottir, Sigridur Kristjansdottir, Rognvaldur Magnusson, Christopher J. Bean, Marco Broccardo, Simone Cesca, Torsten Dahm, Vala Hjorleifsdottir, Banu Mena Cabrera, Claus Milkereit, Nima Nooshiri, Anne Obermann, Roman Racine, Antonio Pio Rinaldi, Vanille Ritz, Pilar Sanchez-Pastor, Stefan Wiemer

**Affiliations:** 1 University of Pisa, Department of Earth Sciences, Pisa, 56126 Italy; 2 ETH-Zurich, Swiss Seismological Service, Zurich, 8092 Switzerland; 3 Iceland GeoSurvey (ISOR), Reykjavik, 108 Iceland; 4 Dublin Institute of Advanced Studies (DIAS), Geophysics Section, Dublin, D04 C932 Ireland; 5 University of Trento, Department of Civil, Environmental and Mechanical Engineering, Trento, 38123 Italy; 6 German Research Centre for Geosciences (GFZ), Section 2.1 Physics of Earthquakes and Volcanoes, Potsdam, 14467 Germany; 7 Reykjavik Energy (OR), Reykjavik, 110 Iceland

**Keywords:** Seismology, Geophysics, Natural hazards

## Abstract

Induced seismicity is one of the main factors that reduces societal acceptance of deep geothermal energy exploitation activities, and felt earthquakes are the main reason for closure of geothermal projects. Implementing innovative tools for real-time monitoring and forecasting of induced seismicity was one of the aims of the recently completed COSEISMIQ project. Within this project, a temporary seismic network was deployed in the Hengill geothermal region in Iceland, the location of the nation’s two largest geothermal power plants. In this paper, we release raw continuous seismic waveforms and seismicity catalogues collected and prepared during this project. This dataset is particularly valuable since a very dense network was deployed in a seismically active region where thousand of earthquakes occur every year. For this reason, the collected dataset can be used across a broad range of research topics in seismology ranging from the development and testing of new data analysis methods to induced seismicity and seismotectonics studies.

## Abstract (summary)

**Table:** 

Measurement(s)	Seismic waveforms (seismograms) • Seismicity (Origin time, location and magnitude of earthquakes)
Technology Type(s)	Seismic stations (velocity sensors) • SeisComP data acquisition and processing system

## Background & Summary

**Fig. 1:**
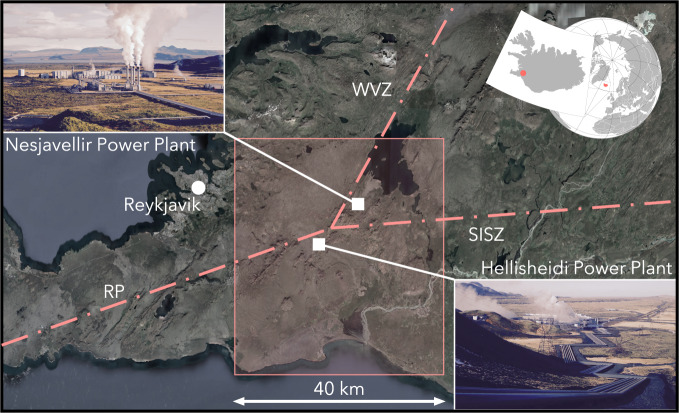
Map of the Hengill area (south-west Iceland) showing the demonstration site of COSEISMIQ (red shaded area), which is a triple junction between the Reykjanes Peninsula oblique rift (RP), the Western Volcanic Zone (WVZ), and the transform-type South Iceland Seismic Zone (SISZ). The white squares are the location of the geothermal power plants.

Over the last decades, the topic of induced seismicity has become increasingly important, in response to the growing concern that industrial activities could induce or trigger damaging earthquakes. The occurrence of felt and damaging events has significant consequences on social acceptance of activities that may produce these events^1^. A recent notable case is the M_*w*_ 5.5 November 2017 Pohang (South Korea) earthquake that has been linked to geothermal energy exploitation operations close to the epicentral area^2–4^. This case highlights the need for new paradigms to manage the risk posed by induced seismicity^4–6^. Within this context, the project COntrol SEISmicity and Manage Induced earthQuakes (COSEISMIQ) aimed to test new generations of real-time induced seismicity management tools^5,6^ using sophisticated real-time seismic monitoring techniques, geomechanical models and seismic hazard and risk analysis methods. The site selected to test these methods is the Hengill region in Iceland (Fig. 1), where geothermal energy has been exploited for electrical power and heat production since the late 1960s^7^. The Hengill geothermal area is located in SW Iceland on the plate boundary between the North American and Eurasian plates. In particular it is located in the triple junction between the oblique spreading-type Reykjanes Peninsula (RP), the Western Volcanic Zone (WVZ), and the transform-type South Iceland Seismic Zone (SISZ) (see Fig. 1). From a seismological point of view this is one of the most active zones on Earth, with many thousands of earthquakes being recorded every year. The Hengill region also hosts the two largest geothermal power plants in Iceland, the Nesjavellir and the Hellisheidi power stations (Fig. 1), thus also the presence of induced seismicity characterizes this area.

The Nesjavellir power plant produces about 120 MW of electricity and supplies hot water to Reykjavik. The production of hot water began in 1990, with electricity production starting from 1998. Re-injection into shallow wells that were drilled and tested in early 2001 started in 2004, with the water entering the rock formation between 400–550 m depth. Since 2000, earthquake activity has mostly been confined to the production and re-injection area of the power plant with several earthquakes up to magnitude 3.5^8^.

The Hellisheidi power plant is the third largest geothermal power plant in the world, producing about 300 MW of electricity and provides heat for domestic heating in Reykjavik^9^. The production began in 2006 and to maintain reservoir pressure, wastewater re-injection in the geothermal reservoir is necessary. Injection operations started in 2006 and increased in the fall of 2011 when a new injection site came into use. The new injection wells were drilled at the periphery of the geothermal field about 1 km northwest of the power plant, targeting the major SSW-NNE faults forming the westernmost part of the graben. Seismic activity occurred during drilling and testing operations of most of the injection wells^10^. The injection at this site received special attention for having triggered several earthquake swarms including two Ml 3.8 earthquakes in October 2011, a few weeks after it was initiated with a flow rate of around 550 L/s^11^. Since this region is also seismically active the problem of discrimination between natural and induced seismicity is also relevant^12^.

In this paper, we announce the release of about 2-years (from 2018/12/01 to 2021/01/31) of high-quality seismic data collected and analyzed during the COSEISMIQ project. The released dataset includes the raw continuous seismic waveforms and seismicity catalogues. The manuscript also describes the methods used to generate the seismicity catalogues. The seismic network comprises stations from a dense temporary deployment comprising broadband and short period sensors operated by the COSEISMIQ project partners, as well as from the background permanent monitoring stations operated by Iceland GeoSurvey (ISOR) and Icelandic Meteorological Office (IMO). All waveform data is distributed via the European Integrated Data Archive (EIDA; http://www.orfeus-eu.org/data/eida/). The catalogues are distributed via ETH-Zurich. All information is openly available through community standard FDSN webservices.

This large dataset is particularly valuable since a very dense network was deployed in a seismically active region where both induced and natural seismicity are occurring. The dataset includes moderate size earthquakes (M_*w*_ > 4). For this reason the collected dataset can be used within a broad range of research topics in seismology. In addition, due to the large number of recorded earthquakes within the selected period (about 12000 manually located events, roughly 16/day) this dataset is very well suited for testing new developed seismic analysis methods and is a perfect playground for the development of data intensive techniques such as waveforms or machine learning based methods.

## Methods

### Data acquisition

**Fig. 2:**
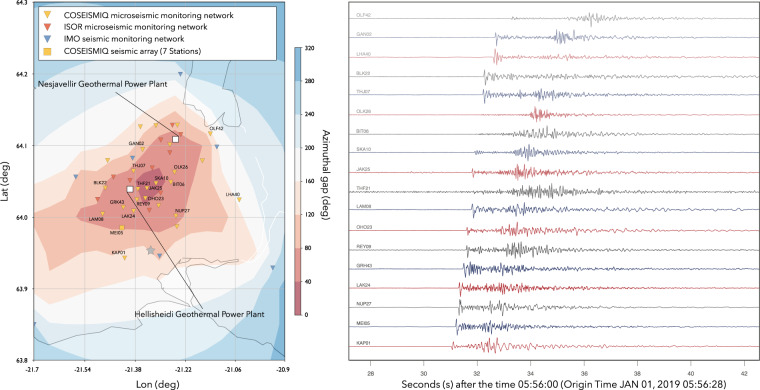
Map of the seismic stations in the Hengill area during the COSEISMIQ experiment. The azimuthal gap for the region using the extended network (COSEISMIQ + ISOR + IMO) is represented by the colour (Left panel). Seismic waveforms (Z component, bandpass filtered between 2 and 50 Hz) from a magnitude ML 1.6 seismic event that occurred on 1.1.2019 at the south edge of the network, Latitude 63.945°N, Longitude −21.327°E at a depth of 7.0 km (Right panel).

Before the COSEISMIQ project the seismicity in the Hengill area had been monitored with about 8 permanent seismic stations of the Icelandic Meteorological Office (IMO) and about 10 permanent stations of a microseismic network managed by the Iceland GeoSurvey (ISOR) for the Reykjavik Energy company. Between November 2018 and August 2021, within the framework of the COSEISMIQ project, the number of seismic stations deployed in the Hengill geothermal field was increased to 44 stations, plus 7 additional stations forming a small aperture seismic array. These were deployed within an area of about 15 × 15 square kilometers and greatly improved the microseismic monitoring capability in the area, with a magnitude of completeness between 0.5 and 1.0. The resulting seismic network is a combination of permanent and temporary networks, composed by short-period (5 s and 1 s) and broad-band (120 s and 60 s) sensors. Figure 2 presents the stations that comprise the seismic monitoring infrastructure of the Hengill region. An initial installation of about 20 temporary seismic stations started in autumn of 2018 (between September and October) and additional stations were added at a later stage. Each seismic station installation consists of: 1) a vault comprised of an insulated buried barrel that houses the sensor and the digitiser and 2) a mast carrying a wind turbine and 2 solar panels for power generation and a cabinet for communication/electrical instruments (the mast is generally 50 m from the vault). Data transmission is performed using WIFI or 4 G network. The variable topographic gradient made the set-up of WIFI range extender antennas necessary. The broadband and short-period seismometers continuously record the seismic data with a sampling rate of 200 Hz (with the exception of IMO stations, sampled at 100 Hz). Seismic data were streamed continuously and in real-time to processing and archival servers at ISOR and ETH-Zurich. The full list of stations, their location, and the hardware deployed, is documented in Table 1. It is important to mention that there were several challenges related to the harsh weather conditions in Hengill region that caused several data gaps in particular during the winter months. The difficulties to perfectly seal the cable entrance to the vault resulted in a handful of submerged stations. Lightening damaged several digitisers. Another challenge was related to strong winds that regularly caused fuse blows at the wind generators.

**Table 1:** Permanent (OR and VI) and temporary (2 C, 4Q) seismic stations in the Hengill area, Iceland.

Network	Station	Latitude (deg)	Longitude (deg)	Elevation (m)	Sensor	Sample Rate (Hz)
2C	BIT06	64.04884	−21.26694	414	STS-2	200
2C	BLK22	64.04066	−21.47562	320	LE-3D5s	200
2C	FAL44	64.10136	−21.27013	250	CMG-6T	200
2C	GAN02	64.09480	−21.35695	295	STS-2	200
2C	GRH43	64.01372	−21.41815	301	CMG-6T	200
2C	JAK25	64.04044	−21.34332	395	LE-3D5s	200
2C	KAP01	63.94302	−21.41364	212	STS-2	200
2C	KAT03	64.07887	−21.16616	346	STS-2	200
2C	LAK24	64.00864	−21.38538	351	LE-3D5s	200
2C	LAM08	64.00454	−21.48423	266	STS-2	200
2C	LHA40	64.02410	−21.04885	111	CMG-6T	200
2C	MEI05	63.98567	−21.42158	310	STS-2	200
2C	MOS29	64.12631	−21.36419	315	LE-3D5s	200
2C	NUP27	64.00175	−21.25100	297	LE-3D5s	200
2C	OHO23	64.02654	−21.34816	378	LE-3D5s	200
2C	OLF42	64.11639	−21.14056	150	CMG-6T	200
2C	OLK26	64.06292	−21.25512	374	LE-3D5s	200
2C	REY09	64.02466	−21.37503	402	STS-2	200
2C	SKA10	64.04740	−21.31417	428	STS-2	200
2C	SKO28	64.12802	−21.31461	345	LE-3D5s	200
2C	STEKK	64.12872	−21.24590	189	LE-3D5s	200
2C	THF21	64.03927	−21.37185	381	LE-3D5s	200
2C	THJ07	64.06443	−21.38608	441	LE-3D5s	200
2C	THU04	63.98698	−21.24774	243	STS-2	200
2C	URD20	64.01628	−21.30566	345	LE-3D5s	200
2C	VAL41	64.07921	−21.46853	230	CMG-6T	200
4Q	MA1	63.98624	−21.42270	299	L-4C-3D	200
4Q	MA2	63.98606	−21.42220	299	L-4C-3D	200
4Q	MA3	63.98589	−21.42170	298	L-4C-3D	200
4Q	MA5	63.98592	−21.42140	297	L-4C-3D	200
4Q	MA5	63.98592	−21.42140	297	L-4C-3D	200
4Q	MA6	63.98613	−21.42110	297	L-4C-3D	200
4Q	MA7	63.98641	−21.42070	297	L-4C-3D	200
OR	GRAFN	64.12855	−21.26174	300	LE-3DliteMkIII	200
OR	HUMLI	64.05130	−21.39710	326	LE-3DliteMkIII	200
OR	HVH	64.00970	−21.33540	381	LE-3DliteMkIII	200
OR	INNST	64.06890	−21.32600	496	LE-3DliteMkIII	200
OR	KOLDU	64.09023	−21.26998	365	LE-3DliteMkIII	200
OR	KRIST	64.02485	−21.50043	362	LE-3DliteMkIII	200
OR	LSKAR	64.03386	−21.29949	393	LE-3DliteMkIII	200
OR	NESJV	64.11550	−21.23512	150	LE-3DliteMkIII	200
OR	SKEGG	64.10792	−21.29883	344	LE-3DliteMkIII	200
OR	SVIN	64.05620	−21.45000	260	LE-3DliteMkIII	200
VI	BJA	63.94590	−21.30258	57	LE-3D5s	100
VI	EDA	64.08258	−21.38898	264	LE-3D5s	100
VI	HEI	64.19978	−21.23604	162	LE1	100
VI	KAS	64.02290	−21.85200	108	LE1	100
VI	KRO	64.09806	−21.11976	147	CMG3ESPC	100
VI	SAN	64.05601	−21.57013	208	LE−3D5s	100
VI	SOL	63.92896	−20.94357	30	LE1	100
VI	VOS	63.85279	−21.70357	8	LE-3D5s	100

This table contains the information of network name (column 1), location (columns 2,3,4), instrument type (column 5) and sampling rate in Hz (column 6).

### Data processing

**Fig. 3:**
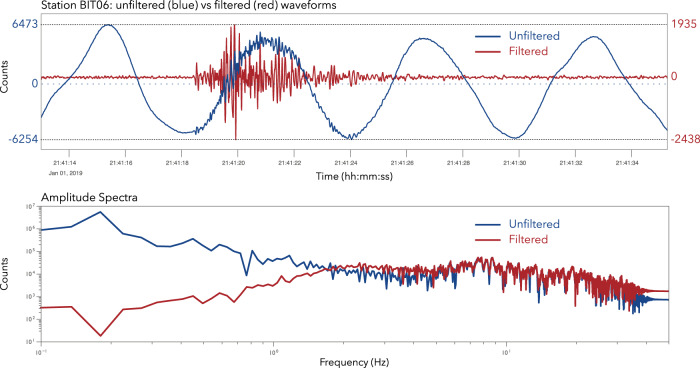
Seismic waveforms and amplitude spectra of the event occurred on 01 January 2019 with magnitude 1.1 recorded by the broadband STS-2 at the station BIT06. The raw, unfiltered waveforms and the amplitude spectrum are shown in blue; the red lines refer to the waveform and amplitude spectrum after filtering between 2–50 Hz.

For the analysis of natural and induced seismicity recorded at the Hengill site in Iceland, we used an optimally tuned SeisComP-based processing server to produce automated seismicity catalogues. SeisComP is a widely used open-source software suite for data acquisition, processing, archiving and visualization of seismic data at global and regional scales^13^, and more recently, also used for microseismic monitoring operations^14^. To create catalogues of seismic events with absolute locations, SeisComP modules for phase detection, phase association, event detection, location, magnitude estimation and quality (score) evaluation are applied in sequential order with the output of each module in general contributing as input for the subsequent module. In a subsequent step, a catalogue of absolute location is used to generate a double difference catalogue using a new SeisComp module, rtDD. In general, SeisComp processing can be performed both in real-time and off-line mode. In this manuscript we only report catalogue information generated from off-line data reprocessing, since the real-time processing was only performed in the last months of the project outside the time-frame of this dataset. Our pipeline starts with the automatic phase picking module using an Akaike Information Criteria (AIC) picker for both P and S phases (although for S ones the picking process starts only after a detection of the P phase)^15^. Phase association and event detection is then performed using the module Scanloc^14^. A refined location is estimated using the Screloc module, which uses the NonLinLoc algorithm^16^ combined with a region-specific minimum 1‐D velocity model^17,18^ developed within the COSEISMIQ project (Table 2)^19^. This model is based on the inversion of about 3000 P-phases and 2200 S-phases manually picked for about 91 seismic events that were recorded during the first 12 months of the COSEISMIQ project. Finally, the local or Richter magnitude (ML) and a location quality score are calculated and the event is added to the catalogue. An important issue we encountered when processing the seismic data from the Hengill area is related to the strong ambient noise contamination of the broadband waveforms that affects local magnitude computation, where a Wood-Anderson filter is applied to the data. Iceland is surrounded by strong oceanic activity that produces an intense environmental noise in the period of 5s–12s^12^. This makes magnitude estimation challenging and, without addressing this issue, for events below Ml 1.0 the energy content of the noise is generally larger than that from the events, even considering the very short hypocentral distances often under 10 km that are typical in leading to an overestimation of station magnitudes if no additional high pass filter is applied to suppress the long period energy. In the catalogues presented here, in order to reduce the impact of the strong microseismic noise, we used a cosine taper filter in the range of 2–50 Hz, implemented within SeisComP. The importance of this filtering process is illustrated for a recording from a earthquake in Fig. 3. Nevertheless, the use of such a filter will lead to underestimation of station magnitudes for larger events because a considerable amount of the event energy can be removed by the filter. The magnitude where this effect becomes significant depends on the corner frequency of the high-pass filter, the 2 Hz corner used here begins to have an effect for local events with Ml above 3.0.

**Table 2:** Velocity model of the Hengill area used to locate seismic events.

Layer depth top (km)	Vp (km/s)	Vs (km/s)
−1.00	2.69	1.63
0.00	3.27	1.69
0.55	3.72	1.89
1.10	4.26	2.15
1.60	4.85	2.77
2.20	5.77	3.35
4.20	6.79	3.80
5.33	7.00	3.90
6.47	7.00	3.98
7.60	7.01	4.06
9.47	7.40	4.06
13.20	7.40	4.07

The model has been extracted from a local tomography study^19^.

A common challenge, particularly in the case of automated catalogues, is providing robust estimates for the quality of an origin. To reduce the number of poor locations or even false detections in the area of interest we adopt a quality score metric (from now on termed ‘quality score’) that has been developed at the Swiss Seismological Service. The quality score, *S*, combines multiple key quality parameters of the origin - the azimuthal gap (*G*, in degrees); the number of P and S phases used, excluding gross outliers (*N*); the origin RMS (*E* in s); the minimum source-station distance (*D* in km); as well as the residual of the pick that corresponds to the 75th percentile (*Q*). The quality score, *S*, is then calculated using the following formula:

1 $$S=-1\left(Q+{\left(\frac{G}{{G}_{cr}}\right)}^{a}+{\left(\frac{E}{{E}_{cr}}\right)}^{b}+{\left(\frac{{N}_{cr}}{0.75N}\right)}^{c}+{\left(\frac{D}{{D}_{cr}}\right)}^{d}\right)$$

*G*_*cr*_, *E*_*cr*_, *Ncr* and *D*_*cr*_ are critical values. The larger a, b, c and d the more “step-wise” the shape. Also note that the score value is negative, a “higher score” is therefore “less negative” and closer to zero. The quality score must be properly tuned by considering the type of application and the area of interest. We optimally tuned the scoring system for the microseismic monitoring operations in the Hengill area. The score threshold and the related parameters are tuned in order to ensure that seismic events with a high-reliable location and relevant for the monitoring purposes (i.e. within the seismic network) are associated with a score ≥ −1.0. On the other hand, seismic events with a score < −5 and at least 10 seismic phases are considered low-quality events with uncertainties of the order of several kilometers and with several outlier picks. Events associated with a score between these two values are considered of intermediate quality and can be associated with small events within the network (M< −5) or events located at the edge of the network. This tuning process is generally performed by following a trial and error optimization scheme, a detailed description on how tune and use the SeisComP quality score module can be found in the official module repository at https://gitlab.seismo.ethz.ch/sed-sc3/evscore/. The equation of the quality score for this specific application is the following:

2 $$S=-1\left(Q+{\left(\frac{G}{225}\right)}^{5}+{\left(\frac{E}{0.15}\right)}^{5}+{\left(\frac{5}{0.75N}\right)}^{5}+{\left(\frac{D}{4}\right)}^{8}\right)$$

**Fig. 4:**
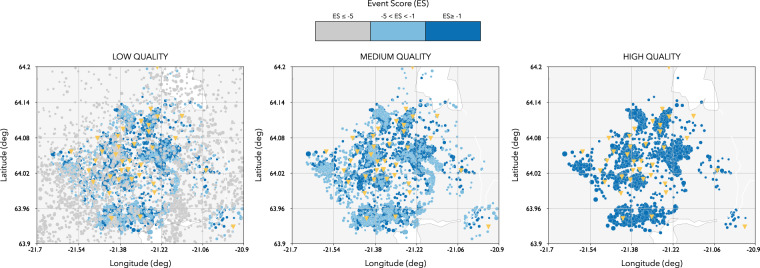
Seismicity location for the **low** (left panel), **medium** (central panel) and **high** (right panel) **quality** catalogues. Event score associated to each event is color coded, in grey events with an event score S≤−5, in light blue the events with events score −5 < S < −1, and in dark blue the events with events score S ≥ −1. Location of seismic stations in yellow.

We use the quality score to create three different absolute catalogues of different quality as illustrated in Fig. 4 and as summarized in Table 3.

**Table 3:** Summary of the different catalogues based on absolute locations.

Catalogue type	Quality Parameters	Number of Events
**low quality catalogue**	number of phases > = 10	about 12000 events
**medium quality catalogue**	number of phases > = −10 and *S* > −5	about 9900 events
**high quality catalogue**	number of phases > = 10 and *S* > −1	about 8500 events

Only the events located within the following geographical region: 63.9° ≤ Latitude (North) ≤ 64.2° and −21.7° ≤ Longitude (East) ≤ −20.9° are contained in each catalogue.

**Fig. 5:**
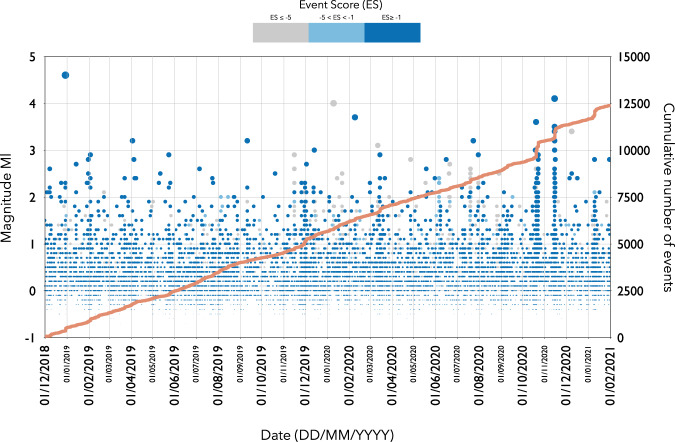
Temporal evolution of seismicity for the **high**, **medium** and **low quality** catalogues. Each event is indicated by a dot. The score associated to each event is color coded, in grey events with an event score S ≤ −5, in light blue the events with events score −5 < S < −1, and in dark blue the events with events score S ≥ −1. The cumulative number of events is indicated by the red line.

Each catalogue only contains the events located within the following geographical region: 63.9° ≤ Latitude (North) ≤ 64.2° and −21.7° ≤ Longitude (East) ≤ −20.9°. The temporal evolution of the seismicity in the Hengill area is illustrated in Fig. 5 which represents both in the magnitude and cumulative number of events versus time for the high, medium and low quality catalogues respectively.

**Fig. 6:**
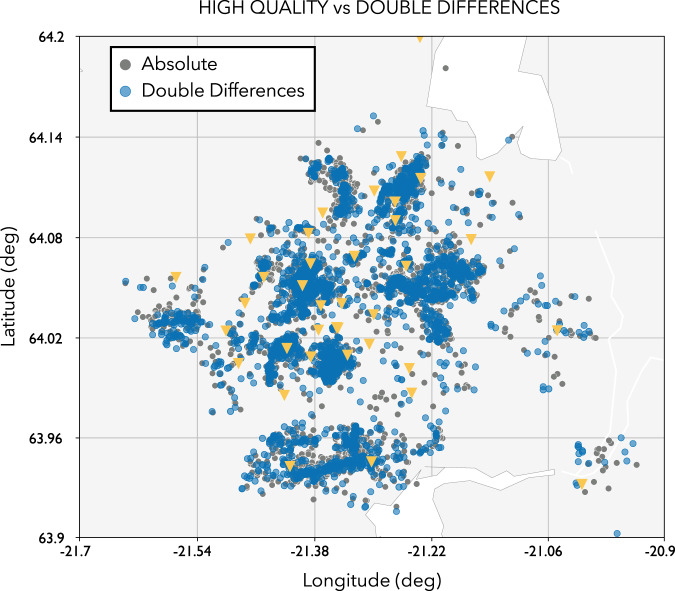
Comparison of locations from the absolute (**high quality**) and double difference catalogues.The location of the seismic stations are indicated by yellow triangles.

In a final step, we further improve the quality of our automated seismic catalogue by using a double-difference relocation algorithm^20,21^ now integrated into SeisComP with the module rtDD^22^. This new module allows both real-time and offline data processing and has been already tested for real-time and offline relocation in Switzerland. In real-time mode, the module adopts the strategy implemented in RT-HypoDD^21^ and it uses waveform cross-correlation and double-difference methods to rapidly relocate new seismic events with high precision using the historical events with accurately known locations (background catalogue). In order to create such a background catalogue, these high-quality events can be relocated using a multi-event double-difference relative relocation procedure (i.e. using rtDD in offline mode). We create a double-difference catalogue using the multi-event procedure restricted only to events in the **high quality** catalogue that have been relocated by using rtDD in offline mode (Fig. 6). Note the significantly enhanced clustering and emergence of lineaments for the double difference catalogue.

## Data Records

The datasets are provided in formats and through services following seismological community standards defined by the International Federation of Digital Seismograph Networks (FDSN, https://www.fdsn.org). Data can be accessed through the the following FDSN web services:

fdsnws-station service to access the station metadata in text and XML format

fdsnws-dataselect service to access the waveform data in miniSEED format

fdsnws-event service to access the event parameters in text and QuakeML format

The continuous raw seismic waveforms are avaialble as binary files in miniSEED format, which is derived from the SEED (Standard for the Exchange of Earthquake Data) data format. While a SEED file consists in both time series values and metadata, the miniSEED format contains only the time series values (binary) and a very limited metadata (identification information). The complete metadata (i.e. station and instrument response information) is stored in a separate file called DATALESS. The metadata describing the stations is available in ascii (i.e. text) and stationXML format (https://stationxml-doc.readthedocs.io/en/release-1.1.0/). The catalogues are available in ascii and quakeMl format (https://quake.ethz.ch/quakeml/). Waveforms, station metadata and seismicity catalogues are available using standard FDSN webservices (https://www.fdsn.org/webservices/). The majority of the temporary COSEISMIQ stations are assigned to a temporary FDSN network code (https://www.fdsn.org/networks) 2 C^23^. For the small aperture array managed by GFZ, the network code is 4Q^24^. The existing stations operated by ISOR use network code OR^25^, and those operated by IMO use network code VI.

Waveform data and its associated metadata from 2 C are permanently hosted at the ETH node of the European Integrated Data Archive (EIDA, https://www.orfeus-eu.org/data/eida/). Data from OR and VI are temporarily hosted at the ETH node, and will be moved to a Icelandic node once it is created. Waveform data and station information can be transparently accessed using the EIDA Federator, which provides direct access to the data irrespective of the actual location of the data. Data from the 4Q network are archived at the GFZ EIDA node. Data at the ETH and GFZ EIDA nodes are stored using the SeisComP Data Structure (SDS, https://www.seiscomp3.org/doc/applications/slarchive/SDS.html), where folders are hierarchically organized by year, network code, station names, and channels. Each miniSEED file is 1-day long is named to uniquely identify the time series. The name of each file includes the network code; the station name; the channel; and the Julian date. The catalogues are available using a persistent ETH endpoint. The Table 4 we show few examples on how to access to the data using the different services. More specifically, the query in Table 4 associated with the **fdsnws-station** can be used to provide a list of all the COSEISMIQ stations. This query returns a text file as the format parameter is set to *text*. The location of the station and the temporal duration of available data is indicated. For the permanent networks OR and VI, only data recorded during the COSEISMIQ project is available, the entire dataset will be made available once an Icelandic EIDA node is created. Information at the network and the channel level can be obtained by setting the parameter *level* equal to network or channel, respectively. Custom requests can be performed by adding or modifying query parameters (more detail in the FDSN webservice site).

**Table 4:** Fdsnws query examples to retrieve: (top) stations metadata using the **fdsnws-station** service, (middle) waverform data using **fdsnws-dataselect** service, and (3) the available catalogues using the **fdsnws-event** service.

**fdsnws-station**	
*address*	http://eida-federator.ethz.ch/fdsnws/station/1/query?
*network*	net = 2C,OR,VI,4Q
*format*	&format = text
*level*	&level = station&nodata = 404
*query*	address+network+format+level
**fdsnws-dataselect**	
*address*	http://eida-federator.ethz.ch/fdsnws/dataselect/1/query?
*network*	net = 2C
*station*	&station = BIT06
*time-period*	&starttime = 2019-01-01T21:41:14&endtime = 2019-01-01T21:41:34&nodata = 404
*query*	address+network+station+time-period
**fdsnws-event**	
*address*	http://coseismiq.ethz.ch:8080/fdsnws/event/1/query?
*time-period*	starttime = 2019-01-01T00:00:00&endtime = 2019-01-01T23:59:59
*contributor*	&contributor = SED_auto_HQ
*format*	&format = text&nodata = 404
*query*	address+time-period+contributor+format

The second query in Table 4 associated with the **fdsnws-dataselect** service describes, with a simple example, the access to waveform data. This request will return the waveform plotted in Fig. 3.

Finally, the last query of Table 4 and associated with **fdsnws-event** service explains how to access the different seismicity catalogues. With this example we retrieve information about the 3 events included in the **high quality** catalogue on the date 1.1.2019 and in text format. By changing the contributor parameter events from the other available catalogues can be retrieved. There are 5 different catalogues that can be requested - SED_auto_LQ, SED_auto_MQ, SED_auto_HQ, SED_auto_HQ_MEDD, ISOR_manual as summarized in the Table 5. These catalogues are also accessible through the figshare repository associated with this paper^26^. The figshare repository also contains shell scripts containing pre-compiled FDSN queries allowing to download both continuous waveforms (full dataset) and event waveforms for each seismic catalogue previously described.

**Table 5:** Catalogues available for download sorted by contributor.

Contributor	Description	Number of events
**SED_auto_LQ**	Low Quality	about 12000
**SED_auto_MQ**	Medium Quality	about 9900
**SED_auto_HQ**	High Quality	about 8500
**SED_auto_HQ_MEDD**	High Quality Double Difference	about 8500
**ISOR_manual**	Manually Reviewed	about 15000

## Technical Validation

**Fig. 7:**
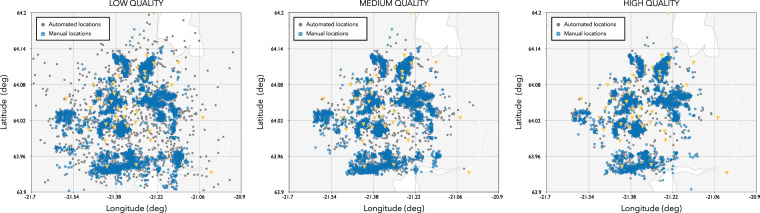
Comparison between the automatic (blue) and ISOR manual (red) seismic events. The comparison is performed for **low** (left panel), **medium** (central panel) and **high** (right panel) quality catalogues.

Quality checking of the recorded waveforms has been performed by looking at data completeness and noise analysis. We analyse catalogue quality and completeness by comparing with the manual ISOR catalogue from the same period. The data completeness for each station of the network (within the entire time-frame of the project) is presented in Figure e.1 (in the electronic supplement) that presents the data availability for each station and the percentage of data completeness. In addition, we calculated the Power Spectral Density (PSD) of the noise at each station of the network. These PSDs are accessible at http://www.seismo.ethz.ch/en/research-and-teaching/products-software/station-information/noise-coseismiq/). We observed that high noise levels affect broadband waveforms within the band frequency 0.1–1.0 Hz (mainly related to the primary and secondary microseisms), hence to correctly determine the magnitude of the seismic events we filtered the waveforms with a bandpass filter in the frequency range 2–50 Hz. To evaluate the overall performance of our automatically generated catalogues, we compare them with the manually reviewed catalogue provided by ISOR. In order to match automatically and manually located events we selected the following matching parameters: 1) origin time difference between two events less than 30 seconds and 2) latitude and longitude difference less than 0.1 degrees. If multiple events satisfy this condition we chose the event pair with the smallest origin-time difference. Figure 7 compares the locations of matching events between each of the **low**, **medium** and **high** quality automated catalogues and the manual catalogue.

**Fig. 8:**
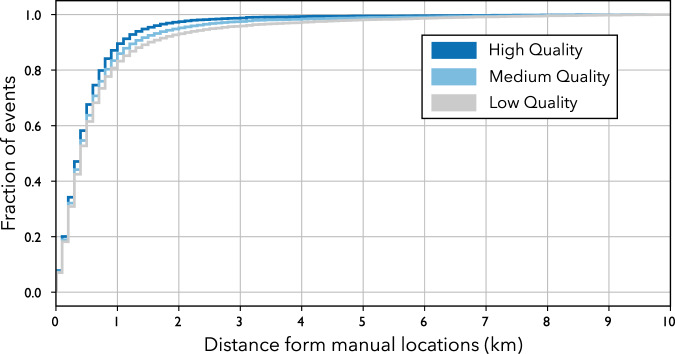
Cumulative distribution of the distances between the automated and manual locations (hypocentral distances) for each matched event in the 3 catalogues. Results for **low** (grey color), **medium** (light blue) and **high** (dark blue) quality catalogues. Note that we plot the cumulative only up to 10 km distance in order to aid visualization, a small fraction of events have location distances in excess of 10 km.

From Fig. 7 it is clear that the **low** quality catalogue includes events with significant location errors, while the **medium** and **high** quality catalogues are more consistent with the manual catalogue provided by ISOR. The average location error (i.e. average difference between the manual and automated locations) for the **low**, **medium** and **high** quality catalogues are 2.6, 1.2 and 0.7 km respectively. Due to large errors on hypocentral coordinates and origin time of the **low** quality catalogue, we were not able to find a match with all the manually inspected earthquakes. An overview of the location errors for each automatic catalogue with respect to matched locations from the manual ISOR catalogue is shown in Fig. 8, showing that for about 80% of the events the hypocentral location difference between the automated (any quality) and matching manual locations is within 1 km. It is important to note that the the **low**, **medium** and **high** quality catalogues are obtained using fully automated procedures and the quality based classification has been performed by filtering the raw catalogue using the quality score and the number of phases as described in the previous section.

## Usage Notes

The Hengill region is characterized by an intense seismic activity, and using the dense seismic network that operated across the 26 months analysed in this manuscript, more than 10,000 events have been detected. The COSEISMIQ seismic network, comprising about 40 stations deployed with an average inter-station distance of about 2 km, is a unique dataset for its genre. The massive number of earthquakes that have occurred in the area, combined with the presence of many seismic sequences characterized by very short inter-event times (about 10 s) makes the analysis of this dataset particularly challenging, and hence is a perfect playground for data intensive techniques such as full-waveform or machine learning based analysis methods^27^. The seismic catalogues (both manual and automated) accompanying this paper can be used as reference to evaluate the performance of newly tested methods. In addition, due to the complex geology of this region, the dataset presented within this paper can be a valuable asset to better studying the natural and induced seismicity of the area. In publishing this dataset (consisting of both continuous raw waveforms and seismicity catalogues) one of our main aim is to provide a baseline for the comparison of fully automated methods for the analysis of seismicity, hence our automatic catalogues have been only sorted by quality score and not manually inspected after their generation. For this reason if not used for the benchmark of newly developed methods, these catalogues should be handled with caution, this is particularly true for the low quality catalogue that includes events with large location errors and false events. The medium and the high quality catalogue (and, of course, the double difference catalogue), on the other hand, are better suited to be used as a starting point for additional seismological analyses (e.g. focal mechanism determination, b-value analysis etc.) or interpretation.

## Supplementary information

Figure e1

## Data Availability

Both the ISOR manual and the automated catalogues were produced using the SeisComP software package. While the core of SeisComP is open source and freely available at http://seiscomp.de, in our analysis we also used the scanloc module which is provided under license by Gempa GmbH http://gempa.de. The evscore and scrtDD modules are open-source and available at https://github.com/swiss-seismological-service.
