# Supplementary material for: Monitoring microseismicity of the Hengill Geothermal Field in Iceland
Source: Sci Data. 2022 May 19;9:220. doi: 10.1038/s41597-022-01339-w (PMC9120172; doi:10.1038/s41597-022-01339-w)
Supplement: Supplementary file 1 — Figure e1 [file 41597_2022_1339_MOESM1_ESM.pdf]

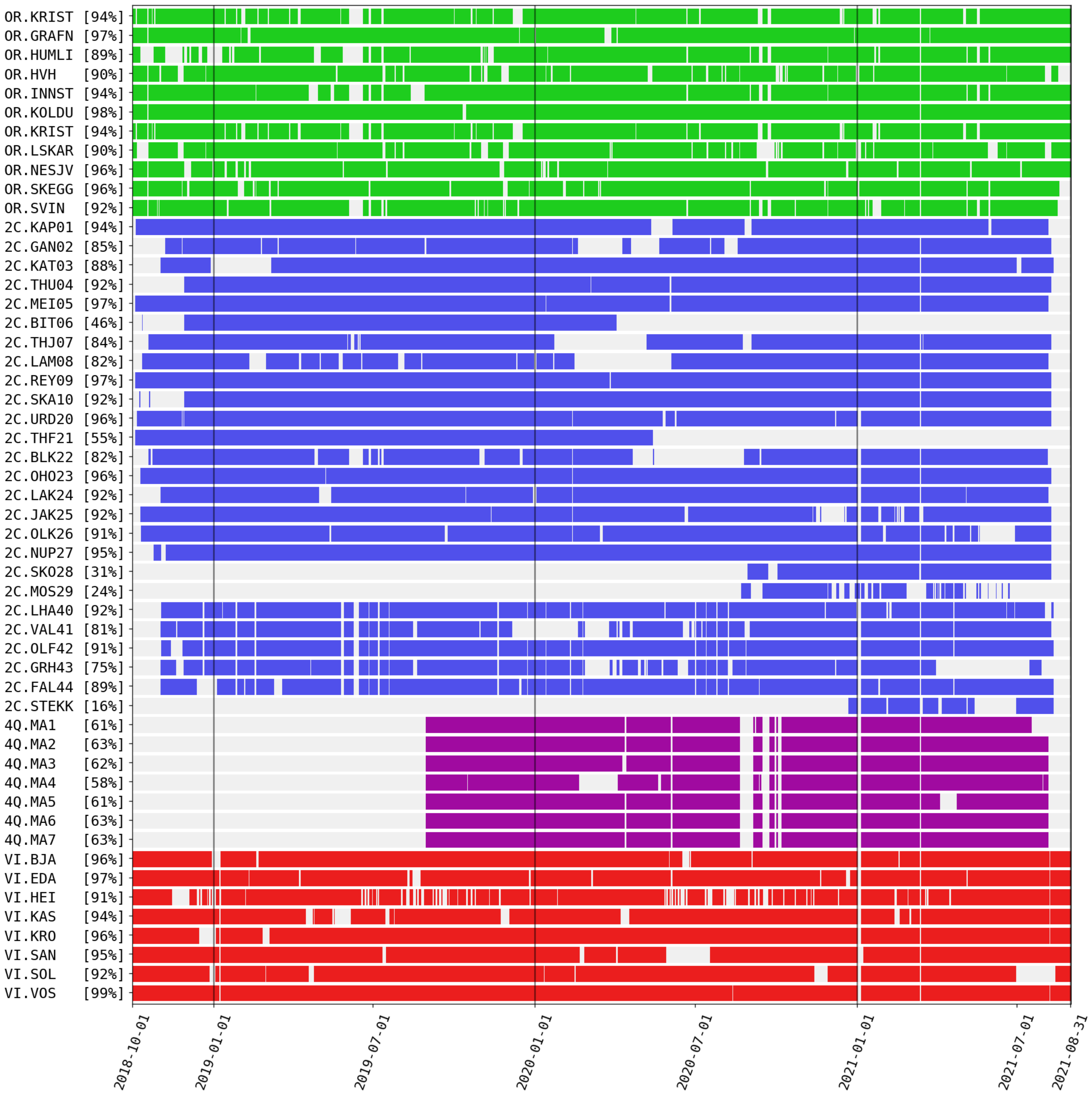

Figure e.1 Data completeness for the Networks OR (Green), 2C (Blue), 4Q (Purple) and VI (Red) covering the entire period to the COSEISMIQ project.
